# The impact of environmental and climate parameters on the incidence and mortality of COVID-19 in the six Gulf Cooperation Council countries: A cross-country comparison study

**DOI:** 10.1371/journal.pone.0269204

**Published:** 2022-07-28

**Authors:** Basema Saddik, Manal A. Awad, Najlaa Al-Bluwi, Amal Hussein, Ankita Shukla, Arwa Al-Shujairi, Hamzah AlZubaidi, Mohamed S. Al-Hajjaj, Rabih Halwani, Qutayba Hamid

**Affiliations:** 1 Department of Family and Community Medicine and Behavioral Sciences, College of Medicine, University of Sharjah, Sharjah, United Arab Emirates; 2 Sharjah Institute of Medical Research, University of Sharjah, Sharjah, United Arab Emirates; 3 Department of Preventive and Restorative Dentistry, College of Dental Medicine, University of Sharjah, Sharjah, United Arab Emirates; 4 College of Pharmacy, University of Sharjah, Sharjah, United Arab Emirates; 5 Clinical Sciences Department, College of Medicine, University of Sharjah, Sharjah, United Arab Emirates; Institute of Oceanology Chinese Academy of Sciences, CHINA

## Abstract

**Background:**

Environmental factors can influence the epidemiological dynamics of COVID-19. To estimate the true impact of these factors on COVID-19, climate and disease data should be monitored and analyzed over an extended period of time. The Gulf Cooperation Council (GCC) countries are particularly lacking in such studies. This ecological study investigates the association between climate parameters and COVID-19 cases and deaths in the GCC.

**Methods:**

Data on temperature, wind-speed and humidity and COVID-19 cases and deaths from the six countries of the GCC were collected between 29/1/2020 and 30/3/2021. Using Spearman’s correlation coefficient, we examined associations between climate parameters and COVID-19 cases and deaths by month, over four different time periods. A two-step cluster analysis was conducted to identify distinct clusters of data using climate parameters and linear regression analysis to determine which climate parameters predicted COVID-19 new cases and deaths.

**Results:**

The United Arab Emirates (UAE) had the highest cumulative number of COVID-19 cases while Bahrain had the highest prevalence rate per 100,000. The Kingdom of Saudi Arabia (KSA) reported the highest cumulative number of deaths while Oman recorded the highest death rate per 100,000. All GCC countries, except the UAE, reported a positive correlation between temperature and cases and deaths. Wind speed was positively correlated with cases in Qatar, but negatively correlated with cases in the UAE and deaths in KSA. Humidity was positively correlated with cases and deaths in Oman, negatively correlated in Bahrain, Kuwait, Qatar and KSA but there was no correlation in the UAE. The most significant predictors in cluster analysis were temperature and humidity, while in the regression analysis, temperature, humidity and wind speed predicted new COVID-19 cases and deaths.

**Conclusion:**

This study provides comprehensive epidemiological information on COVID-19 and climate parameters and preliminary evidence that climate may play a key role in the transmission of the COVID-19 virus. This study will assist decision makers in translating findings into specific guidelines and policies for the prevention and elimination of COVID-19 transmission and infection.

## 1. Introduction

Like many regions across the globe, the Gulf Cooperation Council (GCC) region has been affected by the SARS-CoV-2 virus outbreak. The worldwide pandemic of Coronavirus Disease-19 (COVID-19) was first identified in Wuhan, China in December 2019 [1, 2]. According to Worldometer statistics, as of 30th March 2021, there are over one hundred and thirty million identified cases of COVID-19 worldwide in 221 countries and territories [3]. Moreover, COVID-19 has killed more than 2.5 million people and has forced billions to stay in their homes. The country in which the coronavirus has caused the most devastation is the United States of America (USA) followed by Brazil [4]. The first confirmed case in the GCC was reported by the United Arab Emirates (UAE) on 29 January 2020 [5]. As of 30^th^ March 2021, the United Arab Emirates (UAE) had the highest number of confirmed cases among the GCC [6].

There are a variety of measures being taken to curb the spread of COVID-19 and flatten the epidemic curve, including social distancing, quarantine and vaccination rollouts [7]. Though the outbreak appears to be under control in China [8], it continues to spread worldwide. Human to human transmission has been documented as a major cause of infectious disease outbreaks in the past. A number of environmental factors such as temperature, wind speed and humidity have also contributed to the epidemic dynamics of previous infectious respiratory diseases[9, 10]. As an infectious disease, COVID-19 is no exception. Whether climate might play a crucial role in spreading or containing this pandemic remains unclear. A recent study conducted in 42 countries found that the growth rate of the COVID-19 pandemic should decrease significantly with warmer weather [11]. Increasing temperatures and humidity have also been found to reduce the transmission of COVID-19 and decrease COVID-19 related deaths in China [12, 13]. Comparatively, a study conducted in Norway concluded that although temperature correlated positively with COVID-19 cases, wind speed did not [14]. More recently, a study conducted in Dubai, UAE, reported that higher temperatures, higher solar radiation, and lower humidity were linked to poorer clinical and laboratory outcomes in COVID-19 patients [15].

Despite the reported associations found between climate factors and COVID-19 cases, most studies to date have included less than five months of data in their analysis or have only reported on one country [16–19]. A more recent study found a significant relationship between average temperature and the number of new cases in Bahrain and Qatar, two countries in the GCC, over a three month and subsequent 12 month period [20]. However, further trend analyses are required over a longer time period in order to establish the true impact of climate changes on COVID-19 cases, especially for GCC countries, where such studies are lacking. Furthermore, the geographical locations of countries within the GCC have shown differing ranges of wind speed, solar radiation, and humidity levels, despite the fact that all the countries within the GCC have desertic climates [21]. Therefore, this study aims to provide an overview of the temporal climate changes and their effects on reported cases and deaths related to COVID-19 in the GCC between January 2020 and March 2021. Additionally, this study will examine the association between the number of monthly confirmed cases and deaths for each GCC country and temperature, wind speed and humidity.

## 2. Materials and methods

### 2.1 Study data

COVID-19 data and climate parameters were collected from all six countries in the GCC. These included: Bahrain, Kuwait, Oman, Qatar, Kingdom of Saudi Arabia (KSA) and the United Arab Emirate (UAE). Case data for COVID-19 was collected from the first reported case in each of the six countries, which occurred on 24^th^ February 2020 for Bahrain, Kuwait and Oman, 29^th^ February 2020 for Qatar, 2^nd^ March 2020 for KSA and 29^th^ January 2020 for the UAE. Data were collected until the 30^th^ March 2021.

Publicly available GCC population data and COVID-19 data were retrieved from reliable sources including the official websites of the GCC Ministries of health [22–28] as well as Worldometer which offers global live statistics for COVID-19 [3, 4]. The COVID-19 data included six variables: daily new cases, daily new deaths, daily new recoveries, cumulative number of cases, cumulative number of active cases, and cumulative number of deaths.

Additionally, climate data were also retrieved from publicly available online sources namely the website “Time and Date” which offers live global weather updates [29]. The following climate parameters were collected on a daily basis: maximum temperature (°C), maximum wind speed (km/hr) and maximum humidity (%). The Capital cities of Bahrain, Kuwait, Oman, Qatar and KSA were selected for reporting of climate parameters. However, since Dubai is the most populous Emirate in the UAE, it was chosen instead of Abu Dhabi for the UAE [6].

### 2.2 Statistical analysis

Data were collected and entered into Microsoft Excel, and cleaned and analyzed using IBM SPSS Statistics for Windows, version 25 [30]. Data were tested for normality using the Kolmogorov-Smirnov and Shapiro–Wilk’s tests. All COVID-19 variables and climate parameters in this study were not normally distributed and therefore, were reported as medians with interquartile ranges (Q3-Q1). The six COVID-19 variables collected were compared between the six GCC countries. Descriptive analysis was supported with tables and graphs.

Spearman’s rank correlation coefficient was used to examine the strength of linear association between climate parameters with daily reported cases and deaths of COVID-19 from the first reported case in each country until the 30^th^ March 2021. A more detailed correlation of COVID-19 cases and deaths with climate parameters was also examined by month in each of the six GCC countries. In order to establish a timeframe within which climate may have contributed to incubation of the COVID-19 virus; the three climate parameters were also evaluated over four different timeframes, namely, on the same day, 3 days ago, 7 days ago, and 14 days ago. A description of the strength of the correlation for the absolute value of (r) suggested by Evans (1996) was used while interpreting the correlation [31]. Alpha level of significance was set at p<0.05.

Cluster analysis using a two-step approach was conducted to group data into clusters of similar observations based on the different climate parameters. These parameters included maximum temperature, minimum temperature, wind speed and humidity. Different models were performed to identify the model with best fit. Silhouette measure, which is a measure of cohesion and separation, was used to evaluate model fit, with a value of 0.5 and above indicating a good model. In order to validate the identified model and confirm that the clusters are distinct and vary significantly by the predictor variables, Mann-Whitney U test was used to compare the median values of the predictor variables in the identified cluster groups. Following cluster analysis, outcome variables including the total number of new Covid-19 cases and deaths were compared among the identified clusters.

Multiple linear regression was utilized to determine predictors of new cases and deaths across all GCC countries. The regression model controlled for the three independent variables: temperature, wind speed and humidity. Since the assumption of normality was not met, log transformation of the two dependent variables (new cases and new deaths) was performed. For simpler interpretation, the B coefficient was transferred into percentages using the formula:100*(e^B^-1).

## 3. Results

We collected data from 29^th^ January 2020 to 30^th^ March 2021, a total of 427 days. The Kingdom of Saudi Arabia has the largest population in the GCC (59.4%), followed by the UAE (16.8%), Kuwait (7.3%) Oman (6.8%) Qatar (2.9%) and Bahrain (2.9%) [Table 1].

**Table 1:** COVID-19 cumulative cases, cumulative deaths and cumulative tests performed by GCC countries as of 30^th^ March 2021.

	Cumulative positive cases	Cases per 100,000 population *	Cumulative deaths	Death rate per 100,000 **	Population size	Population proportion	Cumulative tests	Positivity rate***
**Bahrain**	142267	8149	518	30	1745837	2.9%	2834443	5.0%
**Kuwait**	229119	5306	1309	30	4318266	7.3%	2015580	11.4%
**Oman**	158048	3037	1664	32	5204238	6.8%	1550000	10.2%
**Qatar**	179182	6382	289	10	2807805	2.9%	1703244	10.5%
**KSA**	390255	1108	6663	19	35223167	59.4%	12144565	3.2%
**UAE**	458597	4595	1489	15	9980748	16.8%	37269330	1.2%

*Prevalence rate of COVID-19 cases per hundred thousand population = (Cumulative cases/Population size) *100,000

** Deaths rate per hundred thousand population = (Cumulative deaths/Population size) *100,000

***Positivity rate = Cumulative positive cases/ Cumulative tests performed *100

As of 30th March 2021, the UAE had the highest cumulative number of COVID-19 cases (N = 458,597), followed by KSA (N = 390,255) and Kuwait (N = 229,119). However, when these numbers were calculated as rates per 100,000 population, Bahrain had the highest prevalence rate of COVID-19 cases (8,149 per 100,000), whereas KSA had the lowest prevalence rate (1,108 per 100,000). In terms of the cumulative death toll, the highest number was in KSA (N = 6,663) followed by Oman (N = 1,664) and then the UAE (N = 1,489). On the other hand, Oman had the highest death rate due to COVID-19 (32 per 100,000) followed by Kuwait and Bahrain (30 per 100,000). By March 30th 2021, the UAE had conducted the highest number of coronavirus tests followed by the KSA. The positivity rate (which is the percentage of positive test results out of all coronavirus tests performed) was 1.2% and 3.2% for the UAE and KSA respectively. Kuwait had the highest positivity rate (11.4%) [Table 1].

**Fig 1:**
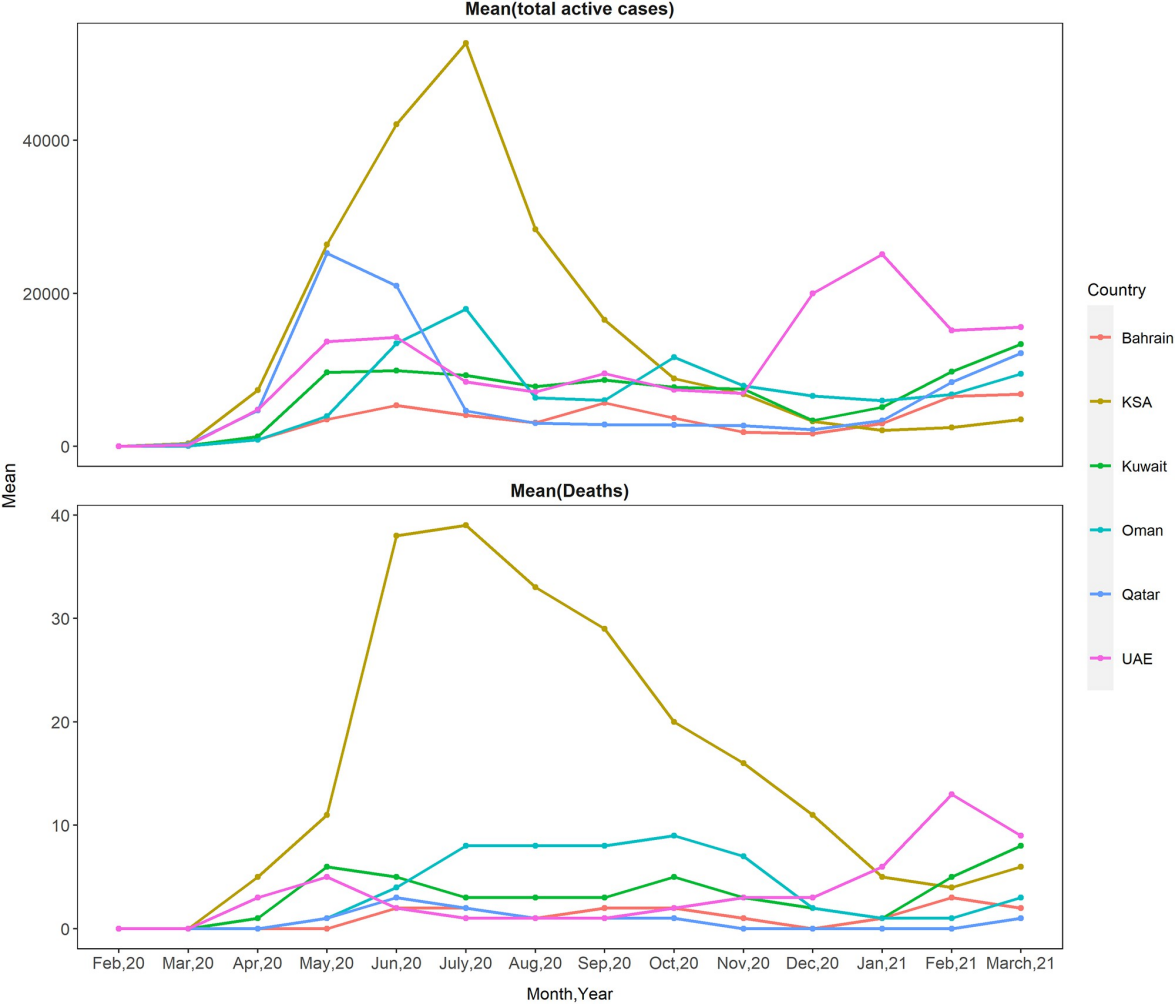
Mean of cumulative COVID-19 active cases and deaths by month in GCC.

Fig 1 presents the peak of mean daily cumulative active COVID-19 cases and daily reported deaths for each month.

There were peaks in mean active cases and deaths in July 2020 (52,690±8,325.1 and 39±10.1) for KSA, February 2021 (6,541±992.4 and 3±1.7) for Bahrain, and March 2021 (13,376±2,543.8 and 8±1.6) for Kuwait. The peaks of active cases in Qatar, Oman and the UAE, were in May 2020 (25,229±7,694.2), July 2020 (17,948±6,103.4) and January 2021 (25,066±1,868.6). The highest deaths occurred in June 2020 (3±1.7) in Qatar, October 2020 in Oman (9±10.6) and February 2021 in the UAE (13±4.0).

Table 2 presents a comparison of COVID-19 data and climate parameters for the six countries within the GCC. The mean, median, minimum and maximum values are presented. For COVID-19 data; the UAE had the highest median of daily new cases (731.0) and KSA had the highest median of daily deaths (11.5). The highest median temperature was recorded in Kuwait, Qatar and UAE (33°C) and the lowest in Bahrain (31°C). The highest median wind speed was recorded in Bahrain (20.0 km/hr.) and the lowest in KSA (14 km/hr.). Humidity was highest in Oman (76%) and lowest in KSA (25%).

**Table 2:** Daily cases and deaths of COVID-19 and climate parameters in GCC countries as of 30^th^ March 2021.

	Bahrain (N = 401)*	Kuwait (N = 401)*	Oman (N = 401)*	Qatar (N = 396)*	KSA (N = 398)*	UAE (N = 427)*	All GCC (N = 2424)*
**Daily new cases**							
Mean (±SD)	354.8 (±232.3)	571.4 (±353.0)	394.3 (±476.4)	449.2 (±445.4)	979.0 (±1118.5)	1070.5 (±1029.3)	641.1 (±756.4)
Median (IQR)	344.0 (343)	582.0 (475)	197.0 (598)	257.0 (290)	406.0 (1173)	731.0 (981)	402.5 (585)
Min-Max	0–1027	0–1613	0–2685	0–2355	0–4919	0–3977	0–4919
**Daily new deaths**							
Mean (±SD)	1.3 (±1.5)	3.3 (±2.8)	4.2 (±6.0)	0.7 (±1.1)	16.7 (±14.0)	3.5 (±4.1)	4.9 (±8.5)
Median (IQR)	1.0 (2)	3.0 (4)	2.0 (7)	0.0 (1)	11.5 (23)	2.0 (4)	2.0 (5.0)
Min-Max	0–7	0–13	0–42	0–7	0–58	0–20	0–58
**Daily new recoveries**							
Mean (±SD)	336.3 (±237.2)	537.1 (±356.1)	381.4 (±648.3)	419.4 (± 650.7)	941.1 (±1168.1)	1085.1 (± 1593.7)	621.8 (±961.4)
Median (IQR)	322.0 (373)	575.0 (484)	166.0 (493)	219.5 (188)	395.5 (1155)	668.0 (1331)	337.0 (574)
Min-Max	0–922	0–1513	0–8386	0–5235	0–7718	0–23885	0–23885
**Daily maximum temperature (c)**							
Mean (±SD)	31.4 (±7.3)	33.6 (±9.9)	31.9 (±5.2)	33.5 (±7.2)	33.0 (±8.3)	33.5 (±6.8)	32.8 (±7.6)
Median (IQR)	31.0 (14)	33.0 (20)	32.0 (9)	33.0 (13)	32.0 (15)	33.0 (13)	32.0 (13)
Min-Max	18–45	15–57	21–45	20–48	14–49	19–47	14–57
**Daily maximum wind speed (km/hr)**							
Mean (±SD)	22.3 (±10.6)	18.4 (±8.9)	21.1 (±16.6)	20.3 (±10.5)	14.8 (±6.0)	19.7 (±9.5)	19.4 (±11.1)
Median (IQR)	20.0 (14)	17.0 (11)	15.0 (7)	17.0 (9)	14.0 (8)	18.0 (6)	17.0 (9)
Min-Max	7–61	3–65	4–86	6–65	4–39	6–74	3–86
**Daily maximum humidity (%)**							
Mean (±SD)	67.7 (±11.3)	51.0 (±23.7)	72.1 (±14.7)	66.5 (±14.6)	30.9 (±20.1)	63.0 (±14.3)	58.6 (±21.9)
Median (IQR)	69.0 (15)	52.0 (43)	76.0 (20)	69.0 (19)	25.0 (28)	64.0 (22)	64.0 (31)
Min-Max	35–94	13–92	26–92	21–98	6–100	24–96	6–100

*N = number of days included in the analysis (from first day of reported case of COVID-19 until 30^th^ March 2021)

**Fig 2:**
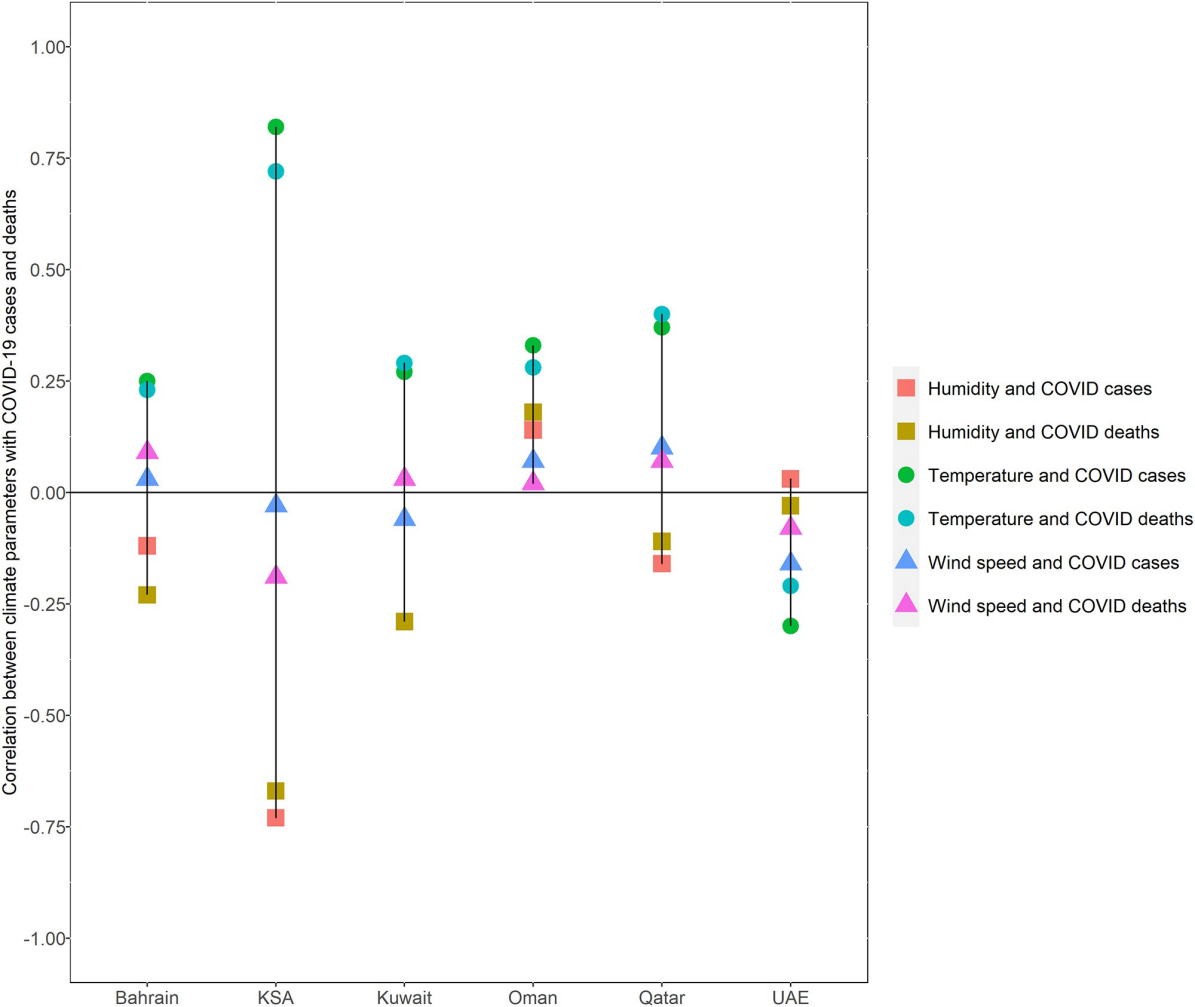
Correlation between COVID-19 cases and deaths with climate parameters in GCC as of 30^th^ March 2021.

Fig 2 displays the results of the Spearman’ s correlation analysis between daily COVID-19 cases and death with climate parameters for the total 427 days within the GCC by country.

A significant positive relationship was found between increases in temperature and new COVID-19 cases and deaths. The strength of this correlation was highest in KSA and ranged from very strong for cases (r (392) = .82, p < .01) to strong for deaths (r (392) = .72, p < .01), followed by weak to moderate correlation in Qatar for cases (r(394) = 0.37, p < .01) and deaths (r(394) = .40, p < .01). Weaker correlations were found for Bahrain (r(399) = .25, p < .01 and r(399) = .23, p < .01), Kuwait (r(399) = .27, p <0.01 and r(399) = .29, p <0.01), and Oman (r(399) = .33, p < .01 and r(399) = .28, p < .01) for both cases and deaths respectively. On the other hand, in the UAE, the results indicated a significant weak inverse correlation between temperature and new COVID-19 cases (r(425) = -.30, p < .01) and deaths (r(425) = -.21, p < .01), demonstrating that as temperature increased in the UAE, the number of new COVID-19 cases decreased.

There was a weak correlation between wind speed and COVID-19 new cases in Qatar (r(394) = .10, p < .05) and UAE (r(425) = -.16, p < .01) and death in KSA (r(392) = -.19, p < .01). An inverse relationship was found for KSA and the UAE. No significant relationships were found in Bahrain, Kuwait and Oman. As for humidity, a significant relationship between COVID-19 cases and deaths in all GCC countries except for the UAE was found. The strength of the correlation for new cases and deaths, respectively, ranged from strong (r(392) = -.73, p < .01 and r(392) = -.67, p < .01) for KSA to weak for Kuwait (r(399) = -.29, p < .01 and r(399) = -.29, p < .01) and very weak for Bahrain (r(399) = -.12, p < .05 and r(399) = -.23, p < .05), Oman (r(399) = .14, p < .01 and r(399) = .18, p < .01), and Qatar (r(394) = -.16, p < .01 and r(394) = -.11, p < .05). Positive correlations were found for Oman whilst inverse correlations were noted for Bahrain, Kuwait, Qatar, and KSA. No significant correlations were found between humidity and cases and deaths for the UAE.

**Fig 3:**
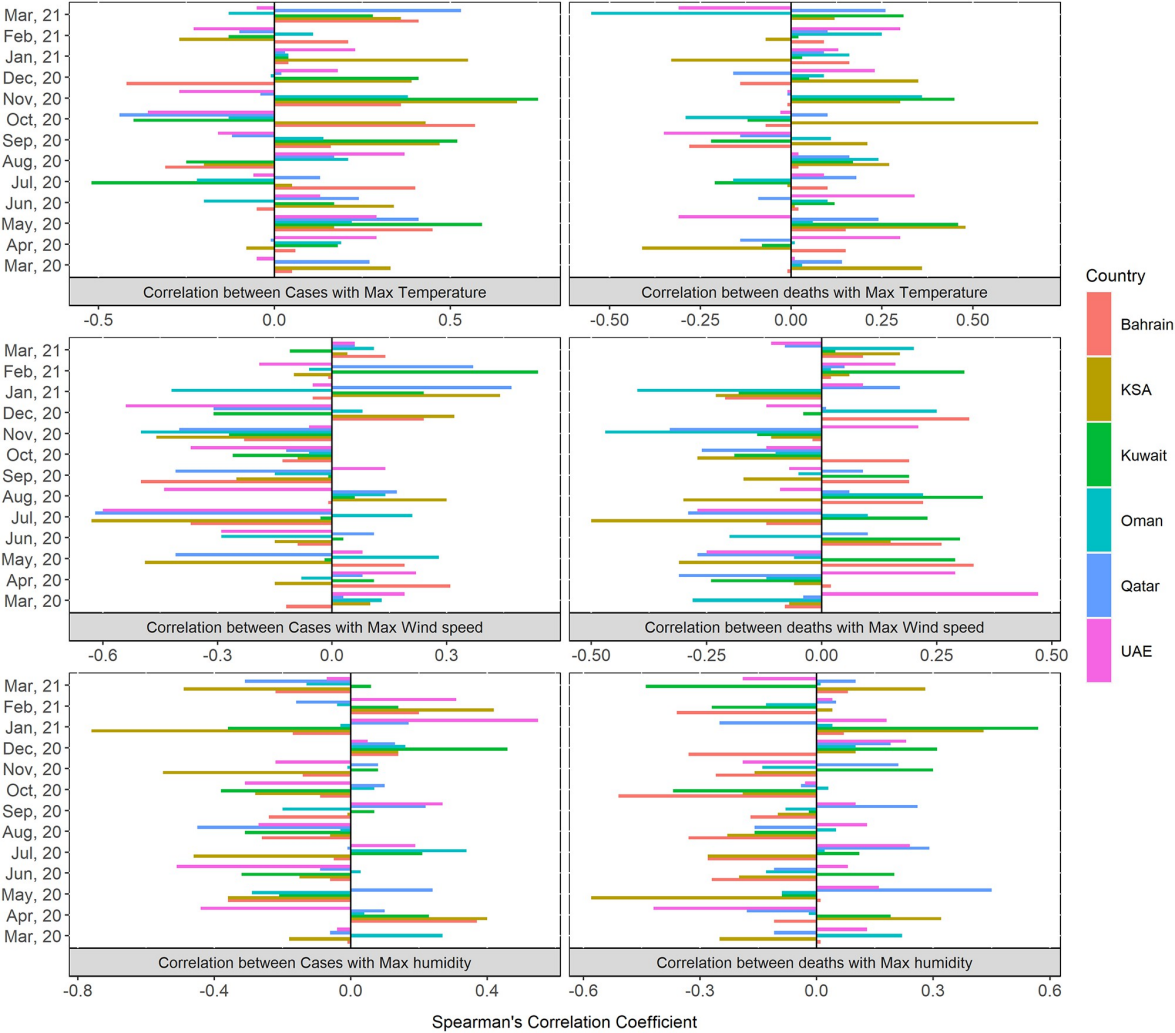
Correlation of cases and death with climate parameters (temperature, windspeed and humidity) by month in GCC countries.

Fig 3 displays correlations by month for each of the six GCC countries. The strength of the correlation between temperature and cases were significantly higher in August 2020 for the UAE (r(29) = .37, p < .05), October 2020 for Bahrain (r(29) = .57, p < .01), November 2020 for Kuwait (r(28) = .75, p < .01) and KSA (r(28) = .69, p < .01) and March 2021 for Qatar (r(28) = .53, p < .01). Higher temperature correlated significantly with more deaths during May 2020 in Kuwait (r(29) = .46, p < .01), October 2020 in KSA (r(29) = .68, p < .01) and March 2021 in Oman (r(28) = -.55, p < .01). No significant correlations were found between temperature per month with cases in Oman and deaths in Bahrain, Qatar and UAE [S1 Table].

Additional trend analysis found significantly negative correlations between COVID-19 cases and wind speed in July 2020 for Qatar (r(29) = -.62, p < .01), KSA (r(29) = -.63, p < .01) and the UAE (r(29) = -.60, p < .01), September 2020 for Bahrain (r(28) = -.50, p < .01), November 2020 for Oman (r(28) = -.50, p < .01) and positive correlations in February 2021 in Kuwait (r(26) = .54, p < .01). In terms of deaths, significant correlations were observed in March 2020 for the UAE (r(29) = .47, p < .01) July 2020 in KSA (r(29) = -.50, p < .01) and November 2020 for Oman (r(28) = -.47, p < .01). However, no significant correlations were found between wind speed and death in Bahrain, Kuwait and Qatar [Fig 3].

Similarly, significant correlations were found between humidity and COVID-19 cases during April 2020 for Bahrain (r(28) = .37, p < .05), June 2020 for UAE (r(28) = -.51, p < .01), August 2020 for Qatar (r(29) = -.45, p < .05), December 2020 for Kuwait (r(29) = .46, p < .05) and January 2021 for KSA (r(29) = -.76, p < .01). There was a significant correlation between deaths and humidity in April 2020 for the UAE (r(28) = -.42, p < .05), May 2020 for Qatar (r(29) = .45, p < .05) and KSA (r(29) = -.58, p < .01), October 2020 for Bahrain (r(29) = -.51, p < .01) and March 2021 for Kuwait (r(28) = -.44, p < .01). There were no significant correlations between humidity and COVID-19 cases and deaths in Oman [Fig 3].

Correlations of new cases with temperature, wind speed and humidity were also evaluated over four different timeframes. Over the four time periods, the correlation between temperature and humidity and the number of new cases did not differ in strength nor direction in all GCC countries except for the UAE where the correlation between number of cases and humidity was highest within 7 days and 14 days. In addition, wind speed did not vary in strength nor direction for cases in Bahrain, Qatar, KSA and the UAE. However, the strength of correlation within 7 days and 14 days was highest in Kuwait and within 3 days in Oman [Table 3].

**Table 3:** Correlation between COVID-19 new cases and climate parameters within four timeframes by GCC country.

Climate parameters	New cases						
	Bahrain (N = 401)	Kuwait (N = 401)	Oman (N = 401)	Qatar (N = 396)	Saudi Arabia (N = 398)	UAE (N = 427)	All GCC (N = 2424)
**Temperature high(c)**							
On the same day	0.25**	0.27**	0.33**	0.37**	0.82**	-0.30**	0.34**
3 days ago	0.26**	0.26**	0.39**	0.36**	0.82**	-0.29**	0.35**
7 days ago	0.22**	0.25**	0.35**	0.31**	0.81**	-0.28**	0.33**
14 days ago	0.22**	0.22**	0.38**	0.23**	0.78**	-0.24**	0.33**
**Wind speed (km/hr)**							
On the same day	0.03	-0.06	0.07	0.10*	-0.03	-0.16**	-0.04
3 days ago	-0.01	-0.06	0.15**	0.08	-0.02	-0.17**	-0.04
7 days ago	0.01	-0.12*	0.07	0.11*	-0.02	-0.17**	-0.05**
14 days ago	-0.01	-0.11*	-0.03	0.12*	0.00	-0.17**	-0.07**
**Humidity (%)**							
On the same day	-0.12*	-0.29**	0.14**	-0.16**	-0.73**	0.03	-0.25*
3 days ago	-0.12*	-0.30*	0.12*	-0.16**	-0.72**	0.00	-0.26*
7 days ago	-0.11*	-0.33*	0.14**	-0.21**	-0.71**	0.02*	-0.26**
14 days ago	-0.10*	-0.32**	-0.10*	-0.19*	-0.63*	0.03*	-0.25**

*significant at the 0.05 level

**significant at the 0.01 level

Two-step cluster analysis revealed a good model with a silhouette measure of cohesion and separation above 0.5 (S1 Fig). The model identified two clusters within the dataset with low temperature, high temperature and humidity being the predictor variables in order of importance. The largest and smallest clusters included 1381(57%) and 1043 (43%) of the data; with a ratio of 1.32. Cluster 1 had a median value of 26.97°C (30.97–24) for the high temperature limit, 18.98 °C (22.08–15.95) for the low temperature limit, and 54.05% (66.94–40.91) for humidity; while in Cluster 2, the median values were 40.07 °C (43.00–37.00), 30.93°C (32.95–27.03), and 24.00% (40.90–12.84), respectively. Results of the Mann-Whitney U test revealed statistical significance when comparing the median values of the three predictors high temperature limit (U = 23352.5, p-value<0.0005), low temperature limit (U = 88770.0, p-value<0.001) and humidity (U = 233404.0, p-value <0.001).

The number of new cases were compared and found to be significantly different between the two clusters. In Cluster 1 and Cluster 2, the median numbers of new cases were 252 (587.5–88) and 574 (957–367) respectively, (U = 412460.0, p-value<0.001). However, the total number of deaths was comparable between the two clusters with median value of 367 (1097–10) in cluster 1 and 308 (558–162) in cluster 2 (U = 701076.5, p-value = 0.262).

The results of the multiple linear regression indicated that temperature, wind speeds and humidity predicted both daily new COVID-19 cases and deaths (S2 Table). For every one-degree Celsius increase in temperature the number of daily new cases increased by 1% while the number of daily new deaths decreased by 0.4% in the GCC. The results also demonstrated that for every one unit increase in wind speed, the number of new cases and new deaths decreased by 0.6% in the GCC. As for humidity, an inverse relationship indicated that for every one unit increase in humidity, the percentage of daily new cases and daily new deaths decreased by 1.1% and 0.9% respectively.

## 4. Discussion

In this ecological study we examined the association between daily cases and deaths of COVID-19 in GCC countries over a period of 427 days with changing climate parameters. Observations from Bahrain, Kuwait, Oman, Qatar and KSA are similar to those reported from Norway [14] and China [32], in which a positive correlation between temperature and COVID-19 cases was observed in Norway and deaths were observed in China. In colder winter climates, such as Norway, warmer temperatures can increase the likelihood of breaking social restriction rules and may also result in people being less strict with COVID-19 precautionary measures. The usually high outdoor temperatures in GCC countries may be associated with an increased likelihood of staying home or socializing indoors, which can contribute to an increase in the incidence of cases. This is especially true since people tend to be more relaxed when they are indoors and do not observe social distance rules or other precautions.

However, the results from our study differ from those of the UAE and Turkey, as well as an analysis of climate comparisons in eight cities [33, 34]. In Turkey and China, an inverse relationship was observed between temperature and the number of reported COVID-19 cases [33, 35]. Similarly, Yu Wu et al revealed that new cases and deaths were negatively related to temperature in 166 Chinese cities [36]. However, a more recent study conducted in the UAE during a shorter period of time than our study, indicated a positive correlation between clinical severity of COVID -19 cases and deaths between January and June 2020 [15]. During the same timeframe (January and June 2020), our study found a positive correlation between temperature and COVID-19 cases and deaths. However, in subsequent timeframes, the direction of the correlation changed [15]. A plausible explanation for this is that the current study provides a comprehensive analysis of recent changes within the pandemic up until March 2021 by evaluating all cases and climate changes over a longer period of time. The contradictory findings observed in the UAE over different time periods may suggest that additional important factors could also play a major role in COVID-19 transmission, such as human factors and behaviours as well as the adherence to precautionary measures. Additionally, the vaccination campaign rollout during the period of data collection may have had an impact on the spread of the virus, as well as, the observed change in the number of cases.

As indicated by reports from countries with different weather conditions, similar to temperature, humidity may also be positively associated with COVID-19 cases [33, 37] which has led researchers to conclude that the combination of higher temperatures and relative humidity may eliminate the viability of the COVID-19 virus [37, 38]. However, our findings, indicated that the number of cases increased as humidity decreased. These findings are consistent with those reported by Al Tamimi et al in Saudi Arabia [39] and Ward et al in Australia [40], in which it was found that a 1% decrease in relative humidity was associated with a 7–8% increase in COVID-19. Additionally, an inverse association was observed between COVID-19 deaths and humidity in the UAE and China [13, 15, 36]. This observation may be explained by the fact that COVID-19 is transmitted primarily through the respiratory route, and infectious elements within the respiratory route remain airborne for longer periods of time due to dry weather [41], which can result in an increase in COVID-19 transmission. Consequently, COVID-19 cases and deaths may decrease with increasing humidity. However, since data are obtained from meteorological recording stations, they do not take into consideration the differences between indoor and outdoor environments [39]. It is worth noting that our findings regarding the effect of humidity on the number of cases reported in the Northern Hemisphere are similar to those reported in the Southern Hemisphere [40], suggesting that humidity might be an important predictor of COVID-19 cases and deaths. Moreover, our findings confirm previous suggestions [40, 41] indicating that when the temperature increases and humidity decreases, the number of cases increases as a result of maintaining an optimal environment for the virus to spread [40–44]. This was further confirmed in our cluster analysis where, in one of the identified clusters, higher temperature and lower humidity were associated with a significant increase in the number of new cases as compared to the second cluster that had lower temperature and higher humidity values.

Consistent with previous reports, our results demonstrated that wind speed was significantly and positively correlated with the number of COVID-19 cases in Qatar [33], and was inversely correlated with the number of cases in the UAE and deaths in KSA. These findings indicate that lower wind speed is associated with less reporting of cases and deaths, which has been previously reported in Saudi Arabia with other viral infections, where low wind speed was correlated with a lower incidence of MERS-CoV cases [17, 39]. Even though these correlations are statistically significant, they are relatively small. Moreover, the cluster analysis revealed that temperature and humidity are more influential in predicting membership into distinct clusters within the data. The effects of different weather conditions on COVID-19 transmission may not be able to be studied independently, and, future research should explore the combined effects of several weather factors on the transmission of COVID-19 [38]. Furthermore, investigating these changes over an extended period of time will help determine the longitudinal effects of temperature and humidity on the transmission of the COVID-19 virus as opposed to spontaneous outbreaks that are more dependent on rapid and unstable climate changes[37].

## 5. Limitations

To the best of our knowledge this is the first study to examine the correlation between COVID-19 cases and deaths and climate parameters over a fifteen month period, by month and at four different timeframes for the GCC. Although this study highlights the impact of climate change on COVID-19 cases and deaths, other important public health factors, such as, lock-down policies, testing capacities, vaccination, social distance, mask wearing and handwashing habits may have a greater impact on the number of COVID-19 cases reported. Moreover, the reported cases and deaths in this study were obtained from publicly available data. Therefore, the real registered number of COVID-19 variables may be higher if they were obtained directly from hospitals. Furthermore, establishing a cause and effect relationship between climate change and COVID-19 cases is premature, since additional important factors need to be considered in order to assess the impact or attributable risk associated with climate change. Considering that the GCC countries share similar potential confounding factors such as lifestyle, economy, response to COVID-19, and culture, the results from this study should not be ignored and will provide important and valuable baseline information for future studies.

## 6. Conclusion

Between 29^th^ January 2020 and 30^th^ March 2021, COVID-19 affected more than 1.5 million people in the GCC and caused around 12 thousand deaths. The UAE had the highest rate of morbidity, while KSA had the highest rate of mortality. The findings from this study will provide researchers with a comprehensive overview of the epidemiology of COVID-19 in the GCC region. Additionally, this study provides a trend and data analysis of climate parameters; temperature, wind speed and humidity, all of which were found to be significant parameters associated with the COVID-19 pandemic in the GCC. These findings provide preliminary evidence that climate has an important role in the transmission of the COVID-19 virus and will assist policy and decision makers to make informed decisions. The impact of these climate factors should be evaluated and integrated into specific guidelines, measures and policies aimed at preventing the transmission of disease.

## Supporting information

S1 Fig Two-step cluster analysis. Two-step cluster analysis revealed a good model with a silhouette measure of cohesion and separation above 0.5.
(TIF)

S1 Table Correlation between COVID-19 cases and deaths with weather components by month in the GCC. (DOCX)

S2 Table Multiple linear regression models for new cases and new deaths. * For simpler interpretation; B coefficient was transferred into percentages using this formula: 100*(e^B^-1) ** Significant if P-Value ≤0.05.
(DOCX)
