# Supplementary figures and images for: The impact of environmental and climate parameters on the incidence and mortality of COVID-19 in the six Gulf Cooperation Council countries: A cross-country comparison study
Source: PLoS One. 2022 Jul 28;17(7):e0269204. doi: 10.1371/journal.pone.0269204 (PMC9333301; doi:10.1371/journal.pone.0269204)

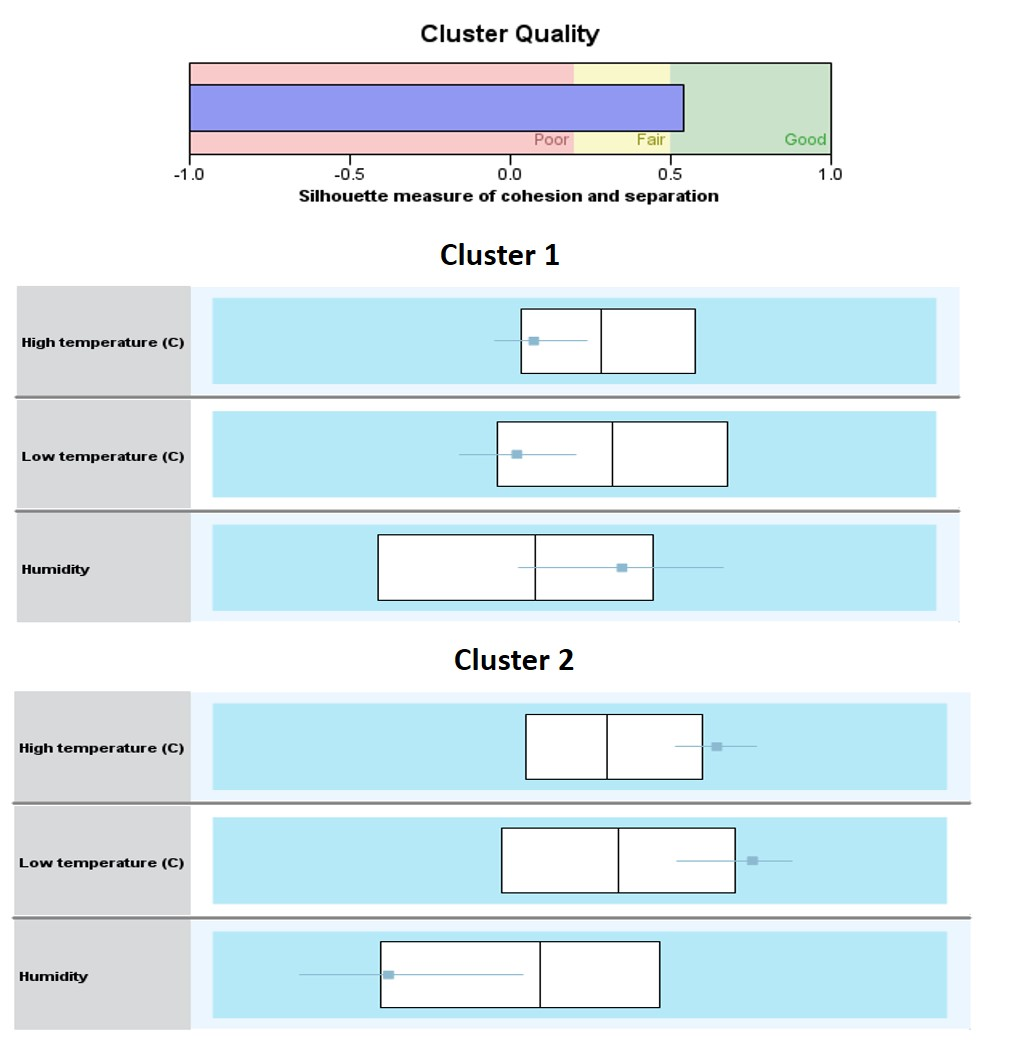

Supplement: S1 Fig — Two-step cluster analysis revealed a good model with a silhouette measure of cohesion and separation above 0.5. (TIF) [file pone.0269204.s001.tif]
