# Supplementary material for: The impact of environmental and climate parameters on the incidence and mortality of COVID-19 in the six Gulf Cooperation Council countries: A cross-country comparison study
Source: PLoS One. 2022 Jul 28;17(7):e0269204. doi: 10.1371/journal.pone.0269204 (PMC9333301; doi:10.1371/journal.pone.0269204)
Supplement: S1 Table — (DOCX) [file pone.0269204.s002.docx]

**The impact of climate and meteorological parameters on COVID-19 cases and deaths in countries of the Gulf Cooperation Council: A cross-country comparison study.**

**Supplementary Table 1: Correlation between COVID-19 Cases and deaths with weather components by month in the GCC**

| **Country/Climate parameters** | | **COVID-19**  **Variables** | **Spearman's rho** | | | | | | | | | | | | | | |
| --- | --- | --- | --- | --- | --- | --- | --- | --- | --- | --- | --- | --- | --- | --- | --- | --- | --- |
|  |  |  | **Jan**  **2020** | **Feb**  **2020** | **Mar**  **2020** | **Apr**  **2020** | **May**  **2020** | **June 2020** | **July**  **2020** | **Aug**  **2020** | **Sep 2020** | **Oct 2020** | **Nov 2020** | **Dec**  **2020** | **Jan 2021** | **Feb**  **2021** | **Mar 2021** |
| **Bahrain** | | | | | | | | | | | | | | | | | |
| Daily maximum temperature | | Cases  Deaths | NA‡  NA | .37  NA | .05  -.01 | .06  .15 | .45*  .15 | -.05  .02 | .40*  .10 | -.31  **-**.02 | .16  -.28 | .57**  -.07 | .36*  -.01 | -.42*  -.14 | .04  .16 | .21  .09 | .41*  .00 |
| Daily maximum wind Speed | | Cases  Deaths | NA  NA | -.03  NA | -.12  -.08 | .31  .02 | .19  .33 | -.09  .26 | -.37*  -.12 | -.01  .22 | -.50**  .19 | -.13  .19 | -.23  -.02 | .24  .32 | -.05  -.21 | -.01  .02 | .14  .09 |
| Daily maximum humidity | | Cases  Deaths | NA  NA | -.15  NA | -.01  .01 | .37*  -.11 | -.36*  .01 | -.06  -.27 | -.05  -.28 | -.26  -.33 | -.24  -.17 | -.09  -.51** | -.14  -.26 | .14  -.33 | -.17  .07 | .20  -.36 | -.22  .08 |
| **Kuwait** | | | | | | | | | | | | | | | | | |
| Daily maximum temperature | | Cases  Deaths | NA  NA | .50  NA | .13  NA | .18  -.08 | .59**  .46** | .17  .12 | -.52**  -.21 | -.25  .17 | .52**  -.22 | -.40*  -.12 | .75**  .45* | .41*  .05 | .04  .03 | -.13  .02 | .28  .31 |
| Daily maximum wind Speed | | Cases  Deaths | NA  NA | .46  NA | .25  NA | .11  -.24 | .03  .29 | -.02  .30 | -.03  .23 | .06  .35 | -.01  .19 | -.26  -.19 | -.27  -.14 | -.31  -.04 | .24  -.18 | .54**  .31 | -.11  .03 |
| Daily maximum humidity | | Cases  Deaths | NA  NA | .09  NA | .39*  NA | .23  .19 | -.21  -.09 | -.32  .20 | .21  .11 | -.31  -.16 | .07  -.02 | -.38*  -.37* | .08  .30 | .46*  .31 | -.36*  .57 | .14  -.27 | .06  -.44* |
| **Oman** | | | | | | | | | | | | | | | | | |
| Daily maximum temperature | | Cases  Deaths | NA  NA | .20  NA | .00  .03 | .19  .01 | .22  .06 | -.20  .10 | -.22  -.16 | .21  .24 | .14  .11 | -.13  -.29 | .36  .36 | -.01  .09 | .04  .16 | .11  .25 | -.31  -.55** |
| Daily maximum wind Speed | | Cases  Deaths | NA  NA | -.40  NA | .13  -.28 | -.08  -.12 | .28  -.06 | -.29  -.20 | .21  .10 | .14  .22 | -.15  -.05 | -.06  -.10 | -.50**  -.47** | .08  .25 | -.42*  -.40* | -.06  .02 | .11  .20 |
| Daily maximum humidity | | Cases  Deaths | NA  NA | .20  NA | .27  .22 | .04  -.02 | -.29  -.09 | .03  -.13 | .34  .02 | -.03  .05 | -.20  -.08 | .07  .03 | -.02  -.14 | .16  .10 | -.03  .04 | -.04  -.13 | -.13  .01 |
| **Qatar** | | | | | | | | | | | | | | | | | |
| Daily maximum temperature | | Cases  Deaths | NA  NA | NA  NA | .27  .14 | -.01  -.14 | .41*  .24 | .24  -.09 | .13  .18 | .17  .16 | -.12  -.14 | -.44*  .10 | -.04  -.01 | .02  -.16 | .03  **.09** | -.10  .10 | .53**  .26 |
| Daily maximum wind Speed | | Cases  Deaths | NA  NA | NA  NA | .03  -.04 | .08  -.31 | -.41*  -.27 | .11  .10 | -.62**  -.29 | .17  .06 | -.41*  .09 | -.12  -.26 | -.40  -.33 | -.31  .01 | .47**  .17 | .37  .05 | .06  -.08 |
| Daily maximum humidity | | Cases  Deaths | NA  NA | NA  NA | -.06  -.11 | .10  -.18 | .24  .45* | -.09  -.11 | -.01  .29 | -.45*  .26 | .22  -.04 | .10  .21 | .08  .21 | .13  .19 | .17  -.25 | -.16  .05 | -.31  .10 |
| **KSA** | | | | | | | | | | | | | | | | | |
| Daily maximum temperature | | Cases  Deaths | NA  NA | NA  NA | .33  .36* | -.08  -.41* | .17  .48** | .34  .01 | .05  -.01 | -.20  .27 | .47**  .21 | .43*  .68** | .69**  .30 | .39*  .35 | .55**  -.33 | -.27  -.07 | .36*  .12 |
| Daily maximum wind Speed | | Cases  Deaths | NA  NA | NA  NA | .10  -.07 | -.15  -.06 | -.49**  -.31 | -.15  .15 | -.63**  -.50** | .30  -.30 | -.25  -.17 | -.09  -.27 | -.46*  -.11 | .32  .00 | .44*  -.23 | -.10  .06 | .04  .17 |
| Daily maximum humidity | | Cases  Deaths | NA  NA | NA  NA | -.18  -.25 | .40*  .32 | -.36  -.58** | -.15  -.21 | -.46**  -.28 | -.06  -.23 | -.02  -.10 | -.28  -.19 | -.55**  -.16 | .14  .10 | -.76**  .43* | .42*  .04 | -.49**  .28 |
| **UAE** | | | | | | | | | | | | | | | | | |
| Daily maximum temperature | | Cases  Deaths | .87  NA | .10  NA | -.05  .01 | .29  .30 | .29  -.31 | .13  .34 | -.06  .09 | .37*  .02 | -.16  -.35 | -.36*  -.03 | -.27  -.01 | .18  .23 | .23  .13 | -.23  .30 | -.05  -.31 |
| Daily maximum wind Speed | | Cases  Deaths | .00  NA | -.12  NA | .19  .47** | .22  .29 | .08  -.25 | -.29  .00 | -.60**  -.27 | -.44*  -.09 | .14  -.07 | -.37*  -.12 | -.06  .21 | -.54**  -.12 | -.05  .09 | -.19  .16 | .06  -.11 |
| Daily maximum humidity | | Cases  Deaths | -.50  NA | .07  NA | -.04  .13 | -.44*  -.42* | .00  .16 | -.51**  .08 | .19  .24 | -.27  .13 | .27  .10 | -.31  -.03 | -.22  -.19 | .05  .23 | .55**  .18 | .31  .04 | -.07  -.19 |
| **‡**NA= not applicable *significant at the 0.05 level **significant at the 0.01 level | | | | | | | | | | | | | | | | | |
|  |  |  |  |  |  |  |  |  |  |  |  |  |  |  |  |  |  |
