# Supplementary material for: The impact of environmental and climate parameters on the incidence and mortality of COVID-19 in the six Gulf Cooperation Council countries: A cross-country comparison study
Source: PLoS One. 2022 Jul 28;17(7):e0269204. doi: 10.1371/journal.pone.0269204 (PMC9333301; doi:10.1371/journal.pone.0269204)
Supplement: S2 Table — * For simpler interpretation; B coefficient was transferred into percentages using this formula: 100*(eB-1) ** Significant if P-Value ≤0.05. (DOCX) [file pone.0269204.s003.docx]

**Table 4. Multiple Linear regression models for new cases and new deaths**

| **Level of regression** | **B (%)*** | **95% CI** | **P-value**** | **B (%)*** | **95% CI** | **P-value*** |
| --- | --- | --- | --- | --- | --- | --- |
| **Temperature high(c)** | 0.010 (1.0) | 0.007, 0.013 | <0.001 | -0.004 (-0.4) | -0.007, -0.002 | 0.002 |
| **Wind speed (km/hr)** | -0.006 (-0.6%) | -0.008, -0.004 | <0.001 | -0.006 (-0.6) | -0.008, -0.005 | <0.001 |
| **Humidity (%)** | -0.011(-1.1%) | -0.013, -0.010 | <0.001 | -0.009(-0.9) | -0.010, -0.008 | <0.001 |

* For simpler interpretation; B coefficient was transferred into percentages using this formula: 100*(e^B^-1)

** Significant if P-Value ≤0.05
